# Comparative Evaluation of Sildenafil Citrate and Estrogen as an Adjuvant Therapy for Treatment of Unexplained Infertility in Women

**DOI:** 10.3390/jpm13050842

**Published:** 2023-05-17

**Authors:** Ahmed E. Altyar, Marian S. Boshra, Ahmed Essam Abou Warda, Sherwet M. Shawkey, Sara Abdallah Mohamed Salem, Rania M. Sarhan, Neven Sarhan

**Affiliations:** 1 Department of Pharmacy Practice, Faculty of Pharmacy, King Abdulaziz University, P.O. Box 80260, Jeddah 21589, Saudi Arabia; 2 Pharmacy Program, Batterjee Medical College, P.O. Box 6231, Jeddah 21442, Saudi Arabia; 3 Clinical Pharmacy Department, Faculty of Pharmacy, Beni-Suef University, Beni-Suef 62511, Egypt; mariansobhy31@yahoo.com (M.S.B.);; 4 Clinical Pharmacy Department, Faculty of Pharmacy, October 6 University, Giza 12585, Egypt; 5 Department of Obstetrics and Gynecology, Faculty of Medicine, Beni-Suef University, Beni-Suef 62511, Egypt; 6 Clinical Pharmacy Department, Faculty of Pharmacy, Misr International University, Cairo 11828, Egypt; nevine.mohamed@miuegypt.edu.eg

**Keywords:** sildenafil citrate, estradiol valerate, clomiphene citrate, ovulation, unexplained infertility, pregnancy rates, endometrial thickness

## Abstract

Background: Uterine blood flow determines endometrial thickness. This study examined how vaginal sildenafil citrate and estradiol valerate altered endometrial thickness, blood flow, and fertility in infertile women. Methods: This study observed 148 infertile women whose infertility was unexplained. Group 1 comprised 48 patients who received oral estradiol valerate (Cyclo-Progynova 2 mg/12 h white tablets) from day 6 till ovulation was initiated with clomiphene citrate. A number of 50 participants in group 2 received oral sildenafil (Respatio 20 mg/12 h film-coated tablets) for 5 days starting the day after their previous menstrual period and finishing on the day they ovulated with clomiphene citrate. Group 3 was the control group, with 50 patients receiving clomiphene citrate (Technovula 50 mg/12 h tablets) ovulation induction from the 2nd to 7th day of cycle. All patients had transvaginal ultrasounds to determine ovulation, follicle count, and fertility. Miscarriage, ectopic pregnancy, and multiple pregnancies were monitored for three months. Results: The three groups’ mean ETs differed statistically at *p* = 0.0004. A statistically significant difference was found between the three groups in terms of the number of follicles, with 69% of patients in group 1 having one and 31% having two or more, 76% of patients in group 2 having one and 24% having two or more, and 90% of patients in the control group having one and 10% having two or more (*p* = 0.038). The clinical pregnancy rates of the three groups were 58%, 46%, and 27%, respectively (*p* = 0.005). The distribution of all side effects was not statistically different between the three groups. Conclusion: It is possible to claim that adding oral estrogen to clomiphene citrate therapy as an adjuvant therapy can improve endometrial thickness and, as a result, increase the pregnancy rates in unexplained infertility compared to sildenafil, especially in cases where the infertility has lasted less than two years. Most people who take sildenafil end up with a mild headache.

## 1. Introduction

Despite the advancements that have been made in assisted reproductive technologies (ARTs), the cumulative success rate of the procedures is still below ideal, with an estimated total pregnancy rate of roughly 30% [1]. Researchers have found that low endometrial receptivity (ER) is a major barrier to successful assisted reproductive technology [2]. Despite extensive research on validating reliable markers, a window of opportunity has yet to be identified for embryo transfer that maximizes implantation success [3]; endometrial thickness (ET) is still regarded as the most accurate surrogate measurement and a crucial implantation component. Consequently, multiple studies found a direct association between low ET (<7 mm) and low success rates of ARTs and medically assisted reproduction (MAR) procedures, including intrauterine insemination (IUI) and in vitro fertilization (IVF) with fresh embryo transfer (fresh-ET) or frozen embryo transfer (frozen-ET) [4,5,6].

During the recent decades, numerous techniques (including hormonal and non-hormonal adjuvants) have been studied with the objective of enhancing ET in women receiving infertility treatments, with mixed outcomes [7,8].

Clomiphene citrate (CC) is one of the non-invasive first-line treatments of infertility which gained great popularity due to its low price, tolerability, and safety profile [9,10,11]. Its antiestrogenic effect plays a crucial role in reducing the estrogen-mediated feedback inhibition resulting in stimulated production of luteinizing hormone (LH) and follicle stimulating hormone (FSH), and this effect represents the core of ovarian stimulation [12]. However, despite the high ratio of ovarian stimulation with CC, it has a high miscarriage rate and low pregnancy rate [13]. This can be attributed to its antiestrogenic action on both the endometrium and cervical mucus [12].

**Figure 1:**
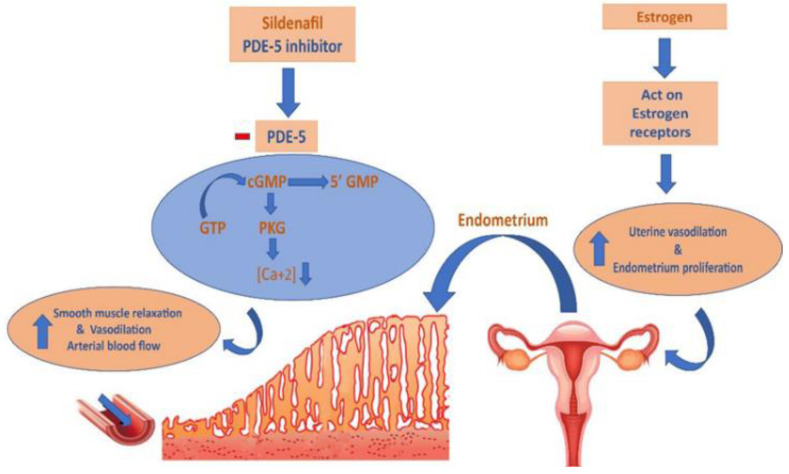
The mechanism of action of sildenafil and estrogen on endometrium thickness. cGMP: cyclic guanosine monophosphate; GTP: guanosine triphosphate.

Sildenafil citrate, a 5-phosphodiesterase inhibitor typically prescribed for males with erectile dysfunction, was found to have a positive effect on endometrial thickening in women undergoing infertility treatments when combined with clomiphene [14]. Sildenafil’s pharmacological impact is predicated on its ability to block the breakdown of cyclic guanosine monophosphate (cGMP), which in turn causes smooth muscle relaxation, vasodilation, and an increase in blood flow to the uterus [14,15,16], as seen in Figure 1. In reality, implantation does not occur in around one-third of euploid blastocyst transfers in women with no obvious endometrial abnormalities [17].

A few randomized controlled studies (RCT) to date have found that estradiol treatment can be given to infertile patients who presented a thin endometrium, in an effort to improve endometrial proliferation, which primarily depends on the flow of blood to the basal endometrium, with a significant increase in the oocyte number [18,19,20]; therefore, more rigorous RCT studies are still needed to determine whether adjuvant treatment with estrogen is beneficial.

Based on these principles, the present study aimed to investigate the efficacy of oral estrogen or sildenafil administration as adjuvant therapy to clomiphene citrate on endometrial thickness, ovulation, and pregnancy rates among women with unexplained infertility.

## 2. Patients and Methods

### 2.1. Setting and Study Design

A prospective study was conducted in the outpatient clinic of the gynecology department of Beni-Suef University Hospital, Beni-Suef, Egypt, between 15 January 2021 and 31 January 2023. The Research Ethical Committee of Beni-Suef University, Faculty of Pharmacy approved carrying out this study with ethical number (REC-H-PhBSU-22022). The study was registered at the Clinical Trials registry (ClinicalTrials.gov; NCT05753098). Each participant gave written informed consent and was informed that participation was voluntary and they might withdraw at any moment. The study followed the Declaration of Helsinki and its amendments.

### 2.2. Patients

The study included 148 women aged between 18 and 40 years with unexplained infertility (primary or secondary) who had a regular menstrual cycle; patent tubes; and husbands with normal semen parameters. The patients were examined prior to the trial between day 16 and day 22 of the previous cycle to rule out any local endometrial lesions. These lesions include endometrial polyps, myomas, thin endometrium, and fluid within the cavity. This was done to ensure that the patients were eligible for the trial as patients with unexplained infertility. Patients with hypotension; cardiovascular, hepatic, and renal diseases; uncontrolled diabetes mellitus; anovulatory infertility; ovarian cysts; pelvic adhesions; hyperprolactinemia; abnormal thyroid functions; multiple uterine fibroids; suspicion of endometriosis and adenomyosis; and abnormal hormonal profile; patients on nitrates; and subjects known to have received any treatment for fertility in the last six months were excluded from the study.

### 2.3. Study Protocol and Follow-Up

Before the commencement of the study, participants underwent ultrasound follow-up visits to confirm that they satisfied the inclusion criteria and to arrive at a diagnosis of unexplained infertility. They were also given an ultrasound to determine the antral follicle count (AFC) on day 3, in addition to having their lab tests for basal FSH and LH levels revised.

A computer created list was used to randomly assign patients to the estrogen therapy in addition to clomiphene citrate group (group 1), sildenafil in addition to clomiphene citrate group (group 2), or control group receiving clomiphene citrate alone (group 3), using sequential numbers.

Group 1 (*n* = 48) received clomiphene citrate 50 mg (Tecnovula^®^) orally twice daily from the 2nd to 7th day of the cycle and estrogen (Cyclopregnova^®^ 2 mg, white tablets, BAYER Schering pharma), one tablet every 12 h from day 6 till triggering of ovulation.

Group 2 (*n* = 50) received clomiphene citrate 50 mg (Tecnovula^®^) orally twice daily from the 2nd to 7th day of the cycle and sildenafil (Respatio^®^ 20 mg film-coated tablets for 5 days) from the last day of menstruation till ovulation.

Group 3/control group (*n* = 50) received clomiphene citrate 50 mg (Tecnovula^®^) orally twice daily from the 2nd to 7th day of the cycle as in the first and second groups, in addition to a placebo tablet.

The transvaginal ultrasonography technique was used for foliculometric analysis. In order to rule out the possibility of ovarian cysts, this procedure was performed on the third day of the cycle. On days 9, 11, and 13, this method was continued until the follicles reached a diameter of 18–22 mm. This was done in order to evaluate the number of follicles as well as the endometrial thickness and any other uterine disease. The mean endometrial thickness of three measurements was determined to exclude any possibility of bias. Triggering ovulation was carried out for all studied patients using 5000 IU of IV human chorionic gonadotropin (hCG) when patients had follicles of 18 mm or more.

Two weeks after ovulation, a blood beta-hCG test was performed to assess pregnancy, and then an ultrasound was utilized to confirm pregnancy. Patients who were unable to conceive after the first cycle of treatment were enrolled for additional cycles of therapy (up to a maximum of three cycles) to begin on day 3 of the following cycle using the same regimen given at enrollment.

### 2.4. Clinical Outcome Measurements

Primary outcome measures included pregnancy rates. Mature follicle counts, ovulation, endometrial thickness, and reported drug-related adverse effects constituted the secondary outcome measures.

### 2.5. Sample Size Calculation

To compute the minimum number of patients required for the two-arm prospective randomized clinical trial, pregnancy outcome proportions from a previous study (P1, P2) among sildenafil/clomiphene versus clomiphene groups of (65% vs. 40%) respectively were used.

Our power calculation showed that a sample size of 55 patients gave us 80% power to detect an effect size f2 of 0.15, given a two-sided tail hypothesis and an alpha level of 0.05. This was the case when we assumed that the alpha level would be 0.05 [21,22].

### 2.6. Statistical Analysis

Categorical data were presented as numbers and proportions and comparisons were performed using chi-square and the Fisher exact test when appropriate. Continuous variables were presented in terms of mean and standard deviation and compared using an unpaired *t*-test for normally distributed data.

Normality testing was conducted employing the Shapiro–Wilk test. Presentation of continuous data showing non-normal distribution was achieved using median and range, and statistical comparison was carried out using the Mann–Whitney U test. Each of the estrogen/clomiphene, sildenafil/clomiphene, and clomiphene groups was subdivided further according to the duration of infertility (less than or equal to 2 years of infertility versus more than 2 years) and an ANOVA test was used to compare outcomes among all the 6 subgroups.

Post hoc statistical analysis was performed, and results were adjusted by Bonferroni correction. Lambda association was used to measure the association between two nominal variables, and the results were expressed in terms of lambda values as a measure for a proportionate reduction in error (PRE). Spearman correlation was used to correlate non-normally distributed numerical variables, and values of rho (ρ) were used to indicate direction and strength of the correlation.

All statistical tests were 2-sided, and tests with a value of *p* < 0.05 were considered statistically significant. All statistical analyses were performed with Statistical Package for Social Sciences (SPSS), version 26 (SPSS Inc., Chicago, IL, USA).

## 3. Results

### 3.1. Baseline Characteristics of Recruited Patients

**Figure 2:**
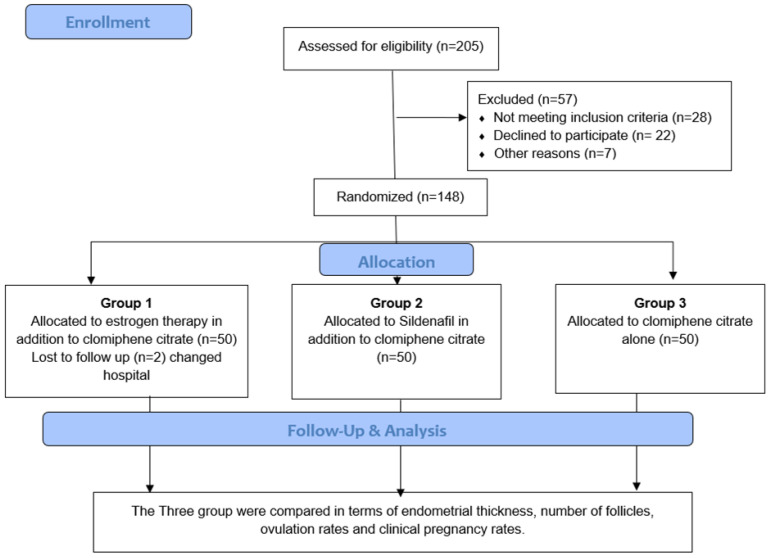
Study flowchart.

A flowchart of all studied subjects is represented in Figure 2. A total of 184 patients were assessed for eligibility and 36 patients were excluded, as 20 of them declined to participate and 16 did not meet the inclusion criteria. After that, the remaining 148 patients were enrolled and randomized in the study. Complete data were obtained for the included patients, and they were randomized through a simple randomization method into three groups according to treatment initiated, with a mean age of 27, 26, and 31 years for the three groups, respectively; however, age categories varied significantly between the three groups, with 12 patients in group 3 above 35 years of age compared to 2 patients in group 1 and none of the patients in group 2 (*p* = 0.003). Moreover, there was no statistically significant difference in BMI among the three groups, as shown in Table 1.

In group 1, 50% of included patients were defined as primary infertile compared to 44% in group 2 and 38% in the control group (*p* = 0.489). The median duration of infertility was 2.3 years in group 1 compared to 2.4 years in group 2 and 2.3 years in the control group (*p* = 0.164), as presented in Table 1. Moreover, patients in group 1 with infertility > 2 years constituted 35% of included patients, compared to 38% in group 2 and 24% in the control group (*p* = 0.283). There was no statistically significant difference between the three groups in either mean year of infertility or proportion of infertility greater than 2 years, as shown in Table 1.

### 3.2. Study Outcomes

The number of follicles showed a statistically significant difference between the three groups, with 69% of the patients having one follicle and 31% having two or more follicles in group 1, compared to 76% of the patients in group 2 having one follicle and 24% having two or more follicles and 90% of the patients in the control group having one follicle and only 10% having two or more follicles (χ^2^ = 4.86, *p* = 0.038), as presented in Table 2. In addition, mean endometrial thickness was significantly different among the three groups with a (*p* = 0.0004) as shown in Table 2. Endometrial thickness was further analyzed among each group’s subpopulations (according to the duration of infertility), as shown in Table 3.

**Figure 3:**
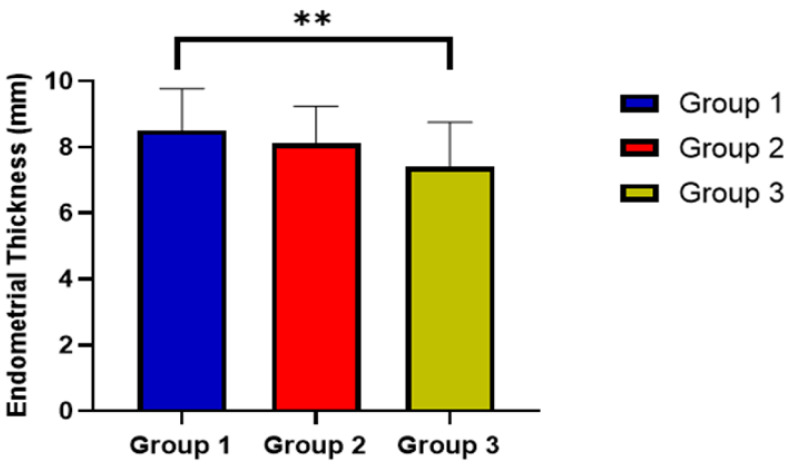
Comparison between the effect of the treatment assigned to the three groups on endometrial thickness. **: indicates significant difference at *p* value < 0.05.

Mean endometrial thickness showed a significantly greater mean (8.6 ± 1.11) in group 1 with infertility duration of less than 2 years compared to other treatment subgroups. Post hoc analysis between each pair of subgroups is shown in Table 4 and Figure 3.

On comparing each subgroup’s mean endometrial thickness, there were statistically significant differences between group 1 with infertility duration ≤ 2 years (8.5 mm) and group 2 (both subgroups, 7.5 and 7.2, respectively). Additionally, a statistically significant difference was found between group 1 with infertility duration > 2 years (8.2) and group 2 with infertility duration of more than 2 years (7.2). Moreover, a statistically significant difference was also found between group 2 with infertility duration ≤ 2 years (7.5) and the control group with infertility duration ≤ 2 years (8.5) and similarly between group 2 with infertility duration > 2 years (7.2) and the control group with infertility duration ≤ 2 years (8.5), as presented in Table 3 and Table 4.

**Figure 4:**
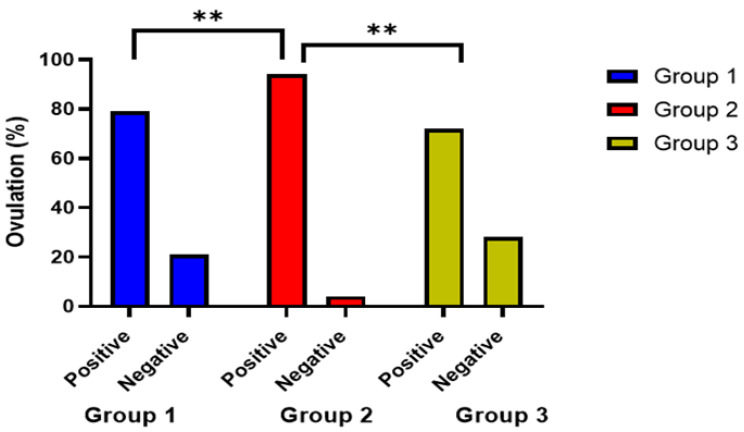
Comparison between the effects of the treatment assigned to the three groups on ovulation rate. **: indicates significant difference at *p* value < 0.05.

**Figure 5:**
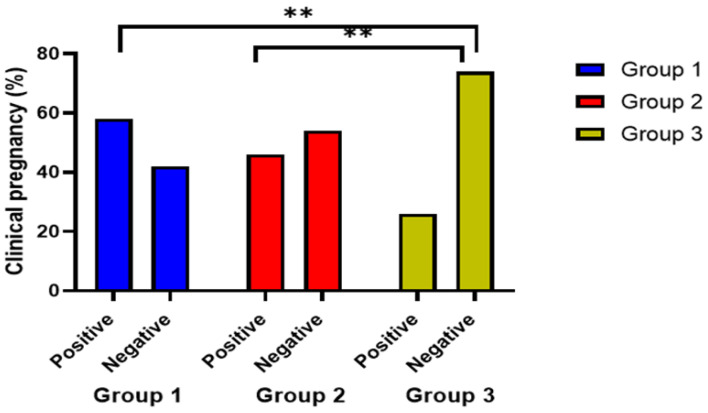
Comparison between the effects of the treatment assigned to the three groups on clinical pregnancy rate. **: indicates significant difference at *p* value < 0.05.

Additionally, the ovulation rate between the three different groups was represented in Figure 4, and the clinical pregnancy rate was statistically different between the three groups, with 58% in group 1 compared to 46% in group 2 and only 26% in the control group (χ^2^ = 10.7, *p* = 0.005), as shown in Table 2. Clinical pregnancy rate showed a significantly greater percentage (41.6%) in group 1 with infertility duration of less than 2 years compared to other treatment subgroups (*p* = 0.003) as shown in Table 5 and Figure 5.

On comparing all side effects distributions among the three groups, there was no statistically significant difference between treatment groups, as shown in Table 6.

### 3.3. Associations

Nominal variables were tested using the lambda test for association. There was no significant association between treatment group and type of infertility, ovulation, clinical pregnancy occurrence, and side effects, as shown in Table 7.

Duration of infertility and BMI were negatively associated with number of follicles in group 2 with a statistically significant difference (ρ (63) = −0.296 and −0.441, *p*-Value = 0.037 and 0.001, respectively), as shown in Table 8. Moreover, age was negatively associated with endometrial thickness in group 1’s difference (ρ (63) = −0.375, *p*-Value = 0.009), as shown in Table 8. Other studied correlations among the three groups are tabulated in Table 8 and show no statistically significant associations.

Multiple linear regression analysis was performed to identify predictors of variability in primary outcomes including endometrial thickness, number of follicles, and clinical pregnancy among the three groups, see Table 9.

## 4. Discussion

Clomiphene citrate (CC) is a standard drug that has been used for a long time for ovulation induction. It is still used as a first-line treatment for women with polycystic ovary syndrome (PCOS) [23]. On the other hand, clomiphene has a number of downsides that are better characterized. Ovulation and pregnancy rates are different after CC treatment from 60–85% versus 10–20%. Miscarriage rates are higher than in the general population [24,25], and 20–25% of PCOS women are clomiphene resistant [26,27]. The anti-estrogenic effect of CC results in a protracted depletion of estrogen receptors, which negatively affects endometrial growth and development, as well as the quality and quantity of cervical mucus. Diverse methods of reducing CC’s estrogenic antagonistic effects have been tried, all with varied outcomes [28,29,30]. Combining either estrogen or sildenafil with clomiphene citrate in the present study aimed at improving rates of pregnancy and endometrial thickness in females with unexplained infertility.

In the present study, the median endometrial thickness in the estrogen/CC group was significantly higher than in the sildenafil/CC and control groups. Such a finding was in accordance with a previous study that showed an increase in endometrium thickness upon using exogenous estradiol in combination with letrozole compared to letrozole alone in the treatment of women undergoing ovulation induction and timed intercourse [31]. This effect was previously explained by Groothuis and colleagues, who investigated the impact of exogenous estradiol on the endometrium of mice and humans. They discovered nuclear translocation in endometrial cells one to three hours after a low dose of estradiol was administered. After three days of estradiol administration, mice had increased endometrial thickness as well as uterine size and weight. The same changes occurred in humans, but they were accompanied by stromal proliferation and required a longer duration of estradiol exposure. They determined that a course of 5 days of estradiol treatment was optimal for generating endometrium of sufficient thickness for implantation [32].

In addition, sildenafil citrate is one of the other adjuvants that, when combined with clomiphene, has shown the potential to have a positive impact on endometrial thickness in women undergoing therapy for infertility [14]. At the level of the endometrium, sildenafil acts by inhibiting the breakdown of cGMP, which results in an increase in the flow of blood via the uterine artery. It also has a beneficial effect on the growth of the endometrium in response to estrogenic stimulation [14,33]. Additionally, it may improve endometrial tolerance to the embryo by lowering the activity of local natural killer cells and promoting healthy embryo implantation, both of which are necessary for successful pregnancy [15]. On the endometrium, the mechanisms by which sildenafil exerts its effects are not completely understood, due to the fact that it is believed to have the effect of promoting implantation both by increasing endometrial thickness [33] and by modulating the immune system’s response [15]. This technique of add-therapy has been examined on a variety of infertile women, including those with a thin endometrium as well as those who do not appear to have any endometrial issues.

However, the ovulation rate was significantly higher among the sildenafil/CC group compared to the other two groups. In accordance with the present study, Benni et al. also illustrated that women with unexplained infertility have experienced an increase in ovulation success and endometrial thickness when oral sildenafil citrate was added to the CC regimen [34].

Moreover, the number of follicles was significantly higher in the estrogen/CC group than in the sildenafil/CC and control groups. Similar to our study results, findings by earlier studies have shown that luteal phase estradiol priming may improve the synchronization of the pool of follicles available for controlled ovarian stimulation, resulting in more favorable responses to controlled ovarian stimulation [35,36]. A few randomized controlled studies (RCT) [35,36,37] to date have found that estradiol treatment was associated with a significant increase in the oocyte number; therefore, more rigorous RCT studies are still needed to determine whether adjuvant treatment with estrogen is beneficial.

The duration of infertility should be taken into consideration during treatment of unexplained infertility. Mean endometrial thickness was significantly greater among those in study group 1 with infertility duration of less than 2 years compared to other treatment subgroups. This means that estrogen as well as sildenafil’s best effect on endometrial thickness can be achieved with women suffering from unexplained infertility for a short period (less than 2 years). This finding has not been reported yet in previous studies and is considered as a proceeding for our study. In addition, the duration of infertility was chosen as the basis for subgroup analysis as numerous investigations confirmed that the pregnancy rate was peculiarly compromised by it [38,39,40,41].

In addition, there was a statistically significant increase in the clinical pregnancy rate among estrogen/CC and sildenafil groups compared to the control group. Our study findings were in contrast to the study by Fetih et al., which reported a considerable rise in endometrial thickness as well as an improvement in uterine blood flow upon addition of sildenafil to CC; however, the rise in the pregnancy rate was not enough to reach statistical significance [42].

In addition, endometrial thickness was negatively associated with age in the estrogen/cc group with a statistically significant difference (*p*-value = 0.009). This means that estrogen/CC will have minimal effect on endometrial thickness and subsequently rate of pregnancy with higher age. Moreover, number of follicles were negatively associated with BMI and duration of infertility (*p*-value = 0.001 and 0.037, respectively), which means that patients with higher BMI and duration of infertility will have a lower number of follicles.

Regarding adverse effects among the three groups, the number of women who experienced adverse effects was high in the study group 2 followed by the study group 1 followed by the control group but with no significant difference. All reported adverse effects were tolerable and no severe cases were reported. Consistent with our findings, Berman et al. illustrated that the most common side effects of sildenafil include headache, blurred vision, flushing, dyspepsia, and nausea [43]. Furthermore, Basson et al. reported that the side effects of sildenafil ranged from mild to moderate in severity and were dosage-related [44]. Moreover, CC contributed to some extent to the reported adverse effects in the present study, as illustrated by Legro et al., who reported that headache, nausea, vomiting, abdominal distension, hot flushes, and disturbed vision are among the adverse effects recorded with the use of CC [45].

## 5. Conclusions

According to the results of our study, oral estrogen, when combined with clomiphene citrate as an adjuvant therapy, can improve the endometrial thickness, leading to an increase in pregnancy rates in women who have unexplained infertility compared with using sildenafil. This is particularly true in cases when the duration of infertility is shorter than two years. Although headaches are the most commonly reported adverse effect of sildenafil medication, there have been no reports of really severe headache.

## Figures and Tables

**Table 1:** Baseline characteristics of the studied patients.

Characteristic	Group 1 (*n* = 48)	Group 2 (*n* = 50)	Control Group (*n* = 50)	*p*-Value
Age				
Age in years (mean ± SD)	27.6 ± 3.84	26 ± 3.72	31.4 ± 4.79	0.230
Age range in years	20–35	19–34	22–39	
Age Categories				
Age from 18 to 24 number (%)	12 (25)	14 (28)	7 (14)	0.003 *
Age From 25 to 29 number (%)	19 (40)	25 (50)	14 (28)	
Age From 30 to 34 number (%)	15 (31)	11 (22)	17 (34)	
Age above 35 number (%)	2 (4)	0 (0)	12 (24)	
Body Mass Index (BMI)				
BMI in Kg/m^2^ (mean ± SD)	23.5 ± 3.38	24.7 ± 2.87	28.6 ± 1.3	0.184
BMI range in Kg/m^2^	18–35	19–34	27–31	
Type of infertility				
Primary infertility number (%)	24 (50)	22 (44)	19 (38)	0.489
Secondary infertility number (%)	24 (50)	28 (56)	31 (62)	
Duration of infertility				
Duration of infertility in years (mean ± SD)	2.3 ± 1.18	2.4 ± 1.17	2.3 ± 1.19	0.164
Infertility duration > 2 years as *n* (%)	17 (35)	19 (38)	12 (24)	0.283

*: indicates significant difference at *p* value < 0.05.

**Table 2:** Clinical outcomes of the study.

Characteristic	Group 1 (*n* = 48)	Group 2 (*n* = 50)	Control Group (*n* = 50)	*p*-Value
Endometrial Thickness and Number of Follicles				
Number of follicles as *n* (%)				0.038 *
One	33 (69)	38 (76)	45 (90)	
Two	13 (27)	11 (22)	3 (6)	
Three	2 (4)	1 (2)	2 (4)	
Endometrial thickness, mean ± SD	8.5 ± 1.27	8.1 ± 1.14	7.4 ± 1.35	0.0004 *
Ovulation				
Positive no. (%)	38 (79)	48 (96)	36 (72)	0.007 *
Negative no. (%)	10 (21)	2 (4)	14 (28)	
Pregnancy				
Positive no. (%)	28 (58)	23 (46)	13 (26)	0.005 *
Negative no. (%)	20 (42)	27 (54)	37 (74)	

*: indicates significant difference at *p* value < 0.05.

**Table 3:** Endometrial thickness comparison among different subpopulations.

Treatment Group	Subgroup	Endometrial Thickness in mm Mean ± SD	*p*-Value
Group 1	Infertility ≤ 2 years	8.6 ± 1.11	0.003 *
	Infertility > 2 years	8.2 ± 1.54	
Group 2	Infertility ≤ 2 years	7.5 ± 1.49	
	Infertility > 2 years	7.2 ± 1.61	
Control group	Infertility ≤ 2 years	8.0 ± 0.98	
	Infertility > 2 years	8.5 ± 1.04	

*: indicates significant difference at *p* value < 0.05.

**Table 4:** A post hoc analysis of endometrial thickness comparison among different subpopulations.

Subgroup	Subpopulation	Adjusted Significance
Group 1; infertility ≤ 2 years	Group 1; infertility > 2 years	0.405
	Group 2; infertility ≤ 2 years	0.001 *
	Group 2; infertility > 2 years	0.001 *
	Group 3; infertility > 2 years	0.061
	Group 3; infertility ≤ 2 years	0.820
Group 1; infertility > 2 years	Group 2; infertility ≤ 2 years	0.065
	Group 2; infertility > 2 years	0.031 *
	Group 3; infertility > 2 years	0.509
	Group 3; infertility ≤ 2 years	0.641
Group 2; infertility ≤ 2 years	Group 2; infertility > 2 years	0.545
	Group 3; infertility > 2 years	0.123
	Group 3; infertility ≤ 2 years	0.029 *
Group 2; infertility > 2 years	Group 3; infertility > 2 years	0.056
	Group 3; infertility ≤ 2 years	0.014 *
Group 3; infertility > 2 years	Group 3; infertility ≤ 2 years	0.258

*: indicates significant difference at *p* value < 0.05.

**Table 5:** Clinical pregnancy comparison among different subpopulations.

Treatment Group	Subgroup	Clinical Pregnancy *n* (%)	*p*-Value
Group 1	Infertility ≤ 2 years	20 (41.6)	0.003 *
	Infertility > 2 years	8 (16.6)	
Group 2	Infertility ≤ 2 years	16 (32)	
	Infertility > 2 years	7 (14)	
Control group	Infertility ≤ 2 years	9 (18)	
	Infertility > 2 years	4 (8)	

*: indicates significant difference at *p* value < 0.05.

**Table 6:** Side effects among different studied groups.

Side Effects No. (%)	Group 1	Group 2	Group 3	*p*-Value
Headache no. (%)	5 (10.4%)	3 (6%)	1 (2%)	0.231
Flushing no. (%)	3 (6.3%)	2 (4%)	2 (4%)	
Blurring vision no. (%)	0 (0%)	6 (12%)	2 (4%)	
GIT upset no. (%)	4 (8.3%)	7 (14%)	1 (2%)	

**Table 7:** Lambda associations between treatment group and study nominal variables.

Variable	Treatment Groups	
	Lambda Value (λ)	*p*-Value
Type of infertility (1ry or 2ry)	0.031	0.723
Ovulation (positive or negative)	0.058	0.114
Clinical pregnancy (yes or no)	0.142	0.117
Overall side effects (yes or no)	0.09	0.166

**Table 8:** Correlation between endometrial thickness and number of follicles in different study groups.

	Endometrial Thickness						Number of Follicles					
	Group 1		Group 2		Control Group		Group 1		Group 2		Control Group	
	ρ	*p*-Value	ρ	*p*-Value	Ρ	*p*-Value	Ρ	*p*-Value	ρ	*p*-Value	ρ	*p*-Value
Age in years	−0.375	0.009 *	0.012	0.935	0.14	0.925	−0.111	0.451	−0.014	0.922	−0.021	0.883
BMI in Kg/m^2^	−0.03	0.983	0.009	0.951	0.096	0.506	0.196	0.182	−0.441	0.001 *	0.018	0.903
Duration of infertility in tears	0.05	0.734	−0.182	0.206	0.121	0.404	−0.071	0.632	−0.296	0.037 *	−0.129	0.371

*: indicates significant difference at *p* value < 0.05.

**Table 9:** Multivariate regression analysis for predictors of primary outcomes among women with unexplained infertility in the three groups.

Predictors	Model 1 Endometrial Thickness		Model 2 Number of Follicles		Model 3 Clinical Pregnancy	
	ꞵ (S.E)	*p*-Value	ꞵ (S.E)	*p*-Value	ꞵ (S.E)	*p*-Value
Age	0.35 (0.57)	0.0001 *	−0.031 (0.01)	0.71	−0.21 (0.08)	0.003 *
Body mass index (BMI)	−0.16 (0.55)	0.002 *	−0.27 (0.13)	0.041 *	−0.174 (0.01)	0.013 *
Infertility duration	−0.15 (0.53)	0.0003 *	−0.15 (0.03)	0.079	0.06 (0.01)	0.37
Endometrial thickness	------	------	0.05 (0.03)	0.070	0.54 (0.02)	0.0001 *
Intercept	1.252	0.005 *	1.739	0.005 *	0.09	0.79
R^2^	0.552		0.15		0.39	

*: indicates significant difference at *p* value < 0.05.

## Data Availability

The data presented in this study are available on request from the corresponding author.
